# Effect of δ-Opioid Receptor Activation on BDNF-TrkB *vs.* TNF-α in the Mouse Cortex Exposed to Prolonged Hypoxia

**DOI:** 10.3390/ijms140815959

**Published:** 2013-07-31

**Authors:** Xuesong Tian, Fei Hua, Harleen K Sandhu, Dongman Chao, Gianfranco Balboni, Severo Salvadori, Xiaozhou He, Ying Xia

**Affiliations:** 1 The Vivan L. Smith Department of Neurosurgery, The University of Texas Medical School at Houston, Houston, TX 77030, USA; E-Mails: xuesong.tian@uth.tmc.edu (X.T.); harleen.k.sandhu@uth.tmc.edu (H.K.S.); dongman.chao@uth.tmc.edu (D.C.); 2 Research Institute of Modern Medicine, The Third Medical College of Soochow University, Changzhou, Jiangsu 213002, China; E-Mail: czhuafei@vip.sina.com; 3 Department of Life and Environment Sciences, University of Cagliari, Cagliari I-09124, Italy; E-Mail: gbalboni@unica.it; 4 Department of Pharmaceutical Sciences, University of Ferrara, Ferrara I-44100, Italy; E-Mail: severo.salvadori@unife.it

**Keywords:** δ-opioid receptor, brain-derived neurotrophic factor (BDNF), TNF-α, hypoxia, neuroprotection

## Abstract

We investigated whether δ-opioid receptor (DOR)-induced neuroprotection involves the brain-derived neurotrophic factor (BDNF) pathway. We studied the effect of DOR activation on the expression of BDNF and other proteins in the cortex of C57BL/6 mice exposed to hypoxia (10% of oxygen) for 1–10 days. The results showed that: (1) 1-day hypoxia had no appreciable effect on BDNF expression, while 3- and 10-day hypoxia progressively decreased BDNF expression, resulting in 37.3% reduction (*p <* 0.05) after 10-day exposure; (2) DOR activation with UFP-512 (1 mg/kg, i.p., daily) partially reversed the hypoxia-induced reduction of BDNF expression in the 3- or 10-day exposed cortex; (3) DOR activation partially reversed the hypoxia-induced reduction in functional TrkB (140-kDa) and attenuated hypoxia-induced increase in truncated TrkB (90-kDa) in the 3- or 10-day hypoxic cortex; and (4) prolonged hypoxia (10 days) significantly increased TNF-α level and decreased CD11b expression in the cortex, which was completely reversed following DOR activation; and (5) there was no significant change in pCREB and pATF-1 levels in the hypoxic cortex. We conclude that prolonged hypoxia down-regulates BDNF-TrkB signaling leading to an increase in TNF-α in the cortex, while DOR activation up-regulates BDNF-TrkB signaling thereby decreasing TNF-α levels in the hypoxic cortex.

## 1. Introduction

Hypoxic/ischemic injury remains the most dreaded cause of neurological disability and mortality. However, neuroprotective therapies against hypoxic/ischemic injury are limited. Our recent studies have demonstrated that the activation of δ-opioid receptors (DOR), which are widely distributed in the mammalian central nervous system (CNS) with a predominance in the cortex, is neuroprotective against hypoxic/ischemic insults in different models including cultured neurons, cortical brain slices and the *in vivo* brain [1–7]. However, the underlying mechanisms of DOR-induced neuroprotection are not yet well understood.

Neurotrophic factors constitute an important element in determining the fate of neurons under hypoxic/ischemic insults. Both *in vivo* and *in vitro* studies have demonstrated BDNF-induced neuroprotection against hypoglycemia, ischemia and hypoxia [8,9]. The BDNF-induced protection is specifically mediated through its tyrosine kinase B (TrkB) receptor [10]. TrkB has two major types of isoforms, *i.e.*, a full-length TrkB receptor that possesses a tyrosine kinase domain, and a truncated isoform that lacks this domain [9]. The truncated isoform is produced under stress [11]. Increased truncated TrkB receptors could contribute to the overall reduced BDNF-TrkB signaling and neuronal injury. Thus, neurotrophin therapy might be directed at blocking the excess production of truncated TrkB [9,12,13]. Previous studies have shown that DOR activation increases the mRNA expression of brain-derived neurotrophic factor (BDNF) in the cortex [14–16], and that this effect is blocked by specific DOR antagonist Naltrindole but not by μ- or κ-opioid receptor antagonists [17]. However, it is unclear whether DOR neuroprotection against hypoxia/ischemia insults involves the regulation of BDNF pathway.

Tumor necrosis factor α (TNF-α), an important pro-inflammatory cytokine, has been shown to exacerbate cerebral ischemic injury [18]. Evidence from an animal study on multiple sclerosis demonstrates that BDNF decreases the expression of TNF-α [19]. In addition, it was observed that the administration of exogenous mature BDNF exerts a neuroprotective effect against TNF-α induced axonal loss through increased phosphorylation of cyclic AMP-response element binding protein (CREB) phosphorylation (pCREB) [20], which is a transcription factor expressed in various CNS neurons. CREB-binding sequences have been identified in different genes including those coding for BDNF. In fact, there is a bidirectional regulation between BDNF and CREB. BDNF can be upregulated by CREB [21]. In addition, BDNF may also induce CREB phosphorylation in rat cortical neurons [22]. Furthermore, DOR agonist [D-Pen^2,5^] enkephalin (DPDPE) produces a dose-dependent increase in the pCREB and this effect is reversed by DOR antagonist Naltrindole [23].

Based on the facts from above mentioned studies, we speculate that the mechanism of DOR neuroprotection against hypoxic injury could involve regulation of BDNF-TrkB signaling pathway and its interaction with the regulation of TNF-α. As an initial step towards testing this possibility, we have performed this investigation to determine if DOR activation affects the expression of BDNF, TrkB and TNF-α in the brain following hypoxic exposure for varying durations.

## 2. Results

### 2.1. Effect of DOR Activation on Cortical BDNF under Hypoxia

**Figure 1:**
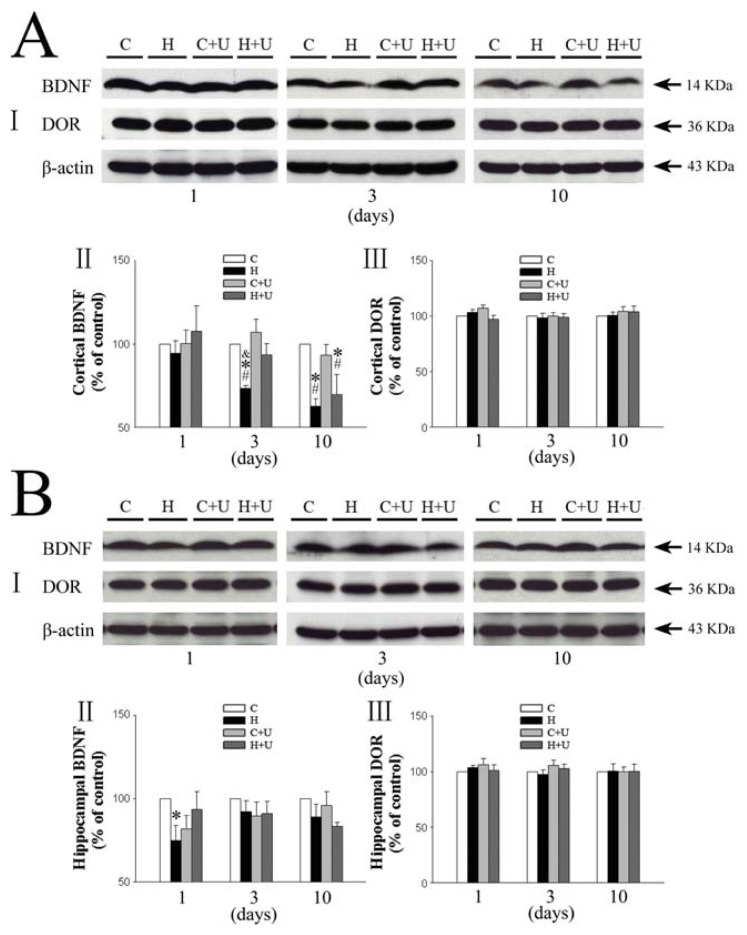
Effect of DOR activation on the expression of BDNF and DOR in the cortex and hippocampus exposed to hypoxia. (**A**) Effect of DOR activation on cortical expression of BDNF and DOR in hypoxia. I, Representative blots of Western blot analysis of the cortical tissues. II, Relative levels of BDNF. III, Relative levels of DOR. C, Normoxic control. H, Hypoxia. C + U (UFP-512), DOR activation with UFP-512 under normoxic conditions. H + U (UFP-512), DOR activation with UFP-512 under hypoxia. *N =* 4, 5 and 6 for 1, 3 and 10 days hypoxic exposure groups, respectively. * *p <* 0.05 *vs.* the control. ^#^*p <* 0.05 *vs.* C + U (UFP-512). ^&^*p <* 0.05 *vs.* H + U. Note that prolonged (3–10 days), but not relatively short-term (1 day), hypoxia significantly reduced the expression of BDNF but not of DOR, Furthermore, DOR activation with UFP-512 partially reversed the hypoxic reduction of BDNF expression; (**B**) Effect of DOR activation on hippocampal expression of BDNF and DOR in hypoxia. I, Representative blots of Western blot analysis of the cortical tissues. II, Relative levels of BDNF. III, Relative levels of DOR. C, Normoxic control. H, Hypoxia. C + U (UFP-512), DOR activation with UFP-512 in normoxic condition. H + U (UFP-512), DOR activation with UFP-512 in hypoxic condition. *N =* 4, 5 and 6 for 1, 3 and 10 days groups, respectively. * *p <* 0.05 *vs.* the control. Note that 1 day hypoxia significantly reduced the expression of BDNF in the hippocampus, while DOR activation partially reversed this hypoxic effect.

We first examined the expression of BDNF and DOR in mouse brain. As shown in Figure 1A, BDNF expression in the cortex slightly decreased after 1 day of hypoxia exposure (94.5% ± 7.6% of the saline control level, *p* > 0.05) (Figure 1A-I and II), and further reduced significantly after subjecting to prolonged hypoxic exposure (73.3% ± 1.8% in 3 days and 62.7% ± 4.6% in 10 days, *p* < 0.05) (Figure 1A-I and II). In contrast, the hypoxia plus UFP-512 (1 mg/kg, i.p., daily) group did not show any significant decrease in the expression of BDNF after 3 days of hypoxic exposure (93.3% ± 6.4% of the saline control level, *p* > 0.05) (Figure 1A-I and II), suggesting attenuation of the hypoxia-induced reduction of BDNF expression by UFP-512 after 3 days of hypoxic exposure. After 10-day exposure, UFP-512 did not significantly affect the hypoxia-induced reduction in BDNF expression (69.5% ± 12.1% of the control), however the expression of BDNF in the UFP-512 group was slightly higher than that of the saline group (62.7% ± 4.6% of the control) though no statistical difference was reached (*p* > 0.05) (Figure 1A-I and II). The DOR expression (Figure 1A-I and III) did not show any significant difference among various groups after prolonged hypoxia (1, 3 and 10 days).

### 2.2. Effect of DOR Activation on Hippocampal BDNF Expression under Hypoxia

We observed a significant decrease in BDNF expression (75.0% ± 9.0% of control, *p <* 0.05) in the hippocampus after 1-day hypoxic exposure as shown in Figure 1B. Also, there was no statistically significant difference (*p >* 0.05) observed after 3 and 10 days of hypoxic exposure (92.3% ± 6.5% and 88.9% ± 7.8% of control, respectively) (Figure 1B-I and II).

However following UFP-512 treatment, we could not see any significant decrease in BDNF levels after 1 day of hypoxic exposure (93.5% ± 10.8% *vs.* 100% of the control level, *p >* 0.05) (Figure 1B-I and II), suggesting that DOR activation reversed the hypoxia-induced reduction of BDNF expression in the hippocampus after hypoxic exposure for 1 day.

Similarly as in the cortex, the DOR expression (Figure 1B-I and III) showed no statistically significant difference after prolonged hypoxia (1, 3 and 10 days).

### 2.3. Effect of DOR Activation on Subcortical and Cerebellar BDNF in Hypoxia

**Figure 2:**
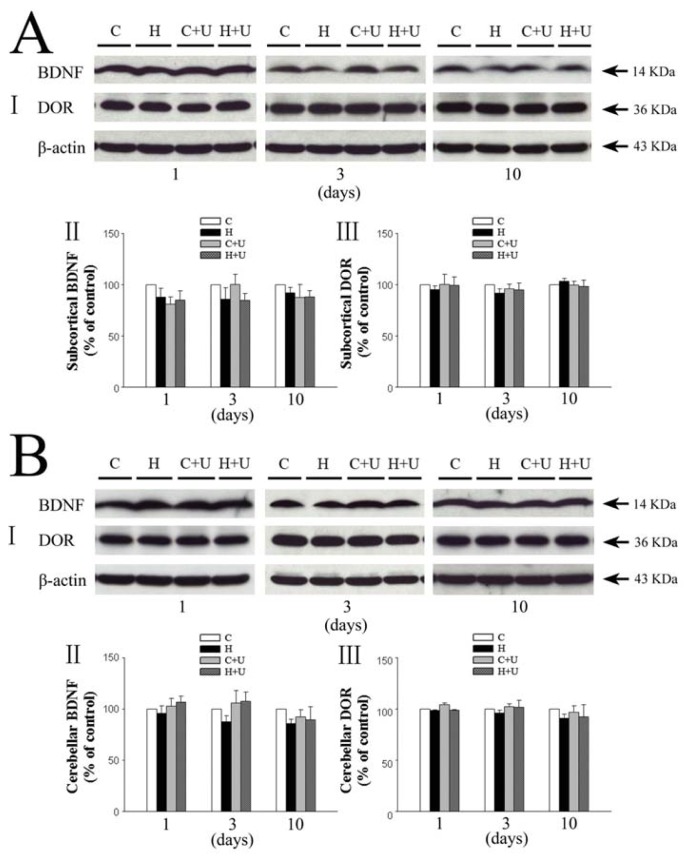
Effect of DOR activation on the expression of BDNF and DOR in the subcortical and cerebellar regions exposed to prolonged hypoxia. (**A**) Effect of DOR activation on BDNF and DOR expression in the subcortical region in hypoxia. I, Representative blots of Western blot analysis of the cortical tissues. II, Relative levels of BDNF. III, Relative levels of DOR. C, Normoxic control. H, Hypoxia. C + U (UFP-512), DOR activation with UFP-512 in normoxic condition. H + U (UFP-512), DOR activation with UFP-512 in hypoxic condition. Note that neither hypoxia nor DOR activation had any significant effect on the expression of BDNF and DOR in the subcortical region; (**B**) Effect of DOR activation on cerebellar expression of BDNF and DOR in hypoxia. I, Representative blots of Western blot analysis of the cortical tissues. II, Relative levels of BDNF. III, Relative levels of DOR. C, Normoxic control. H, Hypoxia. C + U (UFP-512), DOR activation with UFP-512 treatment under normoxic conditions. H + U (UFP-512), DOR activation with UFP-512 treatment under hypoxic conditions. *N =* 4, 5 and 6 for 1, 3 and 10 days groups, respectively. Note that neither hypoxia nor DOR activation had any significant effect on the expression of BDNF and DOR in the cerebellum.

The expression of BDNF in the subcortical tissues of saline hypoxia groups did not show any significant difference (*p >* 0.05) after 1, 3 and 10 days of hypoxic exposure (87.9% ± 8.8% in 1 day, 85.9% ± 11.4% in 3 days and 92.2% ± 5.2% in 10 days; Figure 2A-I and II. The DOR expression (Figure 2A-I and III) also did not show any significant difference after hypoxia exposure for 1–10 days.

The expression of BDNF in the cerebellum of hypoxia groups decreased to 95.9% ± 7.2%, 87.5% ± 6.1% and 86.1% ± 4.0% of the control level, respectively with 1, 3 and 10 days of hypoxia, although the differences were not statistically significant among the groups (*p >* 0.05) (Figure 2B-I and II). The DOR expression in the cerebellum also did not show any significant difference after prolonged hypoxia (1, 3 and 10 days) (Figure 2B-I and III).

### 2.4. Effect of DOR Activation on Cortical TrkB Protein Expression

**Figure 3:**
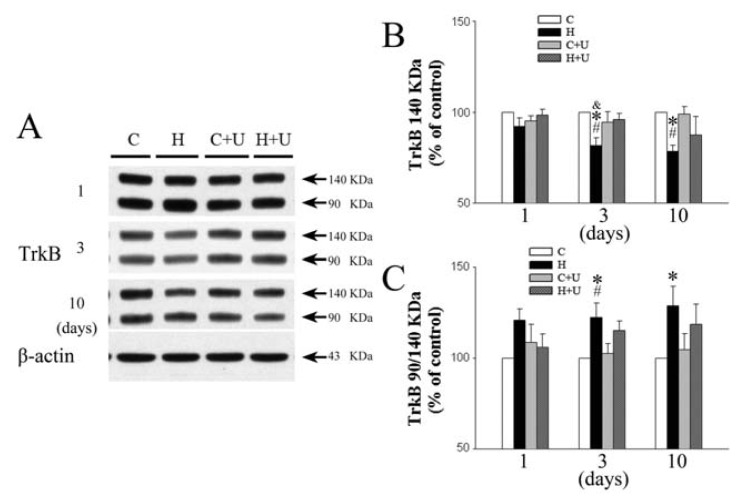
Effect of DOR activation on cortical TrkB expression in hypoxia. (**A**) Representative blots of Western blot analysis of the cortical tissues; (**B**) Relative levels of 140 kDa TrkB. C, Relative levels of 90/140 kDa TrkB; (**C**) Normoxic control. H, Hypoxia. C + U (UFP-512), DOR activation with UFP-512 treatment under normoxic conditions. H + U (UFP-512), DOR activation with UFP-512 treatment under hypoxic conditions. *N =* 4, 5 and 6 for 1, 3 and 10 days groups, respectively. * *p <* 0.05 *vs.* the control. ^#^*p <* 0.05 *vs.* C + U (UFP-512). ^&^*p <* 0.05 *vs.* H + U (UFP-512). Note that 3 or 10 days hypoxia significantly reduced the level of 140 KDa TrkB and increased that of 90/140 kDa TrkB in the cortex, while DOR activation largely reversed these hypoxia-induced changes.

We examined the expression of both full-length and truncated TrkB receptors. As shown in Figure 3, the expression of full-length (140 kDa) TrkB in the cortex of saline hypoxia group was unchanged in response to 1-day hypoxia. However, the expression of 140 kDa TrkB significantly decreased after 3 and 10 days of hypoxic exposure (81.7% ± 4.3% and 78.5% ± 3.3% of control, respectively, (*p <* 0.05)) (Figure 3A,B). In contrast, the hypoxia plus UFP-512 (1 mg/kg) hypoxia group did not show any significant decrease in the expression of 140 kDa TrkB after 3 days of hypoxia (96.0% ± 3.4% of the control, *p >* 0.05) suggesting that UFP-512 attenuated the hypoxia-induced reduction of 140 KDa TrkB expression after 3 days of hypoxic exposure. Although UFP-512 did not significantly affect the hypoxia-induced reduction in 140 kDa TrkB expression after 10 days of hypoxic exposure, the expression of 140 kDa TrkB in UFP-512 (87.5% ± 10.1% of the control, *p <* 0.05) was higher compared to that without hypoxic exposure (78.5% ± 3.3% of the saline control level, *p <* 0.05) (Figure 3A,B).

The production of truncated isoform of TrkB (90 kDa) receptors is induced in response to an injury [11]. Our results indicated hypoxia significantly increased the level of 90 kDa TrkB compared with 140 kDa TrkB after 3–10 days of hypoxic exposure, reaching the levels of 122.3% ± 7.9% and 128.7% ± 10.8% over the normoxic control, (*p <* 0.05) (Figure 3A,C). In contrast, the UFP-512 (1 mg/kg) hypoxia group showed a partial decrease in the expression of 90 kDa TrkB after 3 and 10 days of hypoxic exposure (115.3% ± 5.2% and 118.6% ± 11.1% of the saline control level, *p >* 0.05) suggesting that UFP-512 attenuated the hypoxia-induced truncation of 140 kDa TrkB.

### 2.5. Effect of DOR Activation on Cortical TNF-α Protein Expression

**Figure 4:**
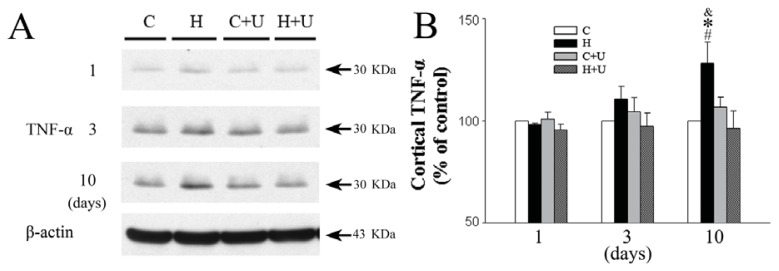
Effect of DOR activation on cortical TNF-α expression in hypoxia. (**A**) Representative blots of Western blot analysis of the cortical tissues; (**B**) Relative levels of TNF-α. C, Normoxic control. H, Hypoxia. C + U (UFP-512), DOR activation with UFP-512 in normoxic condition. H + U (UFP-512), DOR activation with UFP-512 in hypoxic condition. *N =* 4, 5 and 6 for 1, 3 and 10 days groups, respectively. * *p <* 0.05 *vs.* the control. ^#^*p <* 0.05 *vs.* C + U (UFP-512). ^&^*p <* 0.05 *vs.* H + U (UFP-512). Note that 10 days hypoxia significantly increased the level of TNF-α, while DOR activation completely inhibited such increase.

We further examined whether hypoxia affected the expression of TNF-α in the cortex. The expression of TNF-α in saline hypoxia group was unchanged in response to 1 day of hypoxic exposure (98.2% ± 0.7% of control, *p >* 0.05) (Figure 4A,B), but the expression of TNF-α gradually increased after 3 days (110.7% ± 6.1%, *p >* 0.05), and was significantly upregulated after 10 days of hypoxic exposure (128.1% ± 10.4%, *p <* 0.05) (Figure 4A,B). In contrast, the UFP-512 (1 mg/kg) hypoxia group showed a significant decrease in the expression of TNF-α after 10 days of hypoxia (96.4% ± 8.4% of the saline control level, *p <* 0.05) (Figure 4A,B) suggesting that DOR activation attenuates the hypoxia-induced upregulation of TNF-α expression.

### 2.6. Effect of DOR Activation on Cortical CD11b Protein Expression

**Figure 5:**
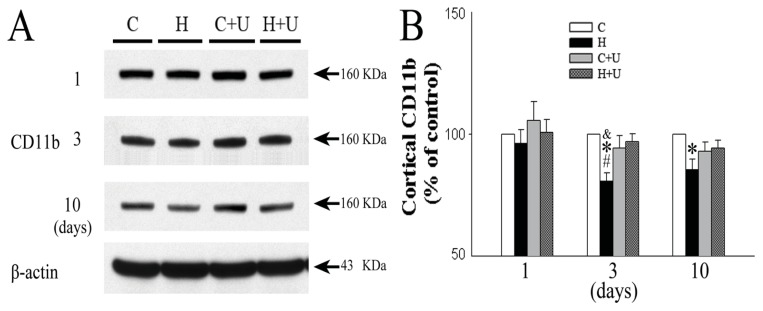
Effect of DOR activation on cortical CD11b expression in hypoxia. (**A**) Representative blots of Western blot analysis of the cortical tissues; (**B**) Relative levels of CD11b. C, Normoxic control. H, Hypoxia. C + U (UFP-512), DOR activation with UFP-512 treatment under normoxic conditions. H + U (UFP-512), DOR activation with UFP-512 treatment under hypoxic conditions. *N =* 4, 5 and 6 for 1, 3 and 10 days groups, respectively. * *p <* 0.05 *vs.* the control. ^#^*p <* 0.05 *vs.* C + U (UFP-512). ^&^*p <* 0.05 *vs.* H + U (UFP-512). Note that 3 or 10 days hypoxia significantly reduced the level of CD11b, while DOR activation with UFP-512 reversed this hypoxic reduction.

We further examined whether hypoxia affected the expression of CD11b in the cortex. The expression of CD11b in saline hypoxia group was unchanged in response to 1 day of hypoxic exposure (96.1% ± 5.7% of the saline control level, *p >* 0.05), but gradually decreased after 3 days (80.8% ± 3.4% of the saline control level, *p >* 0.05), and 10 days of hypoxic exposure (85.4% ± 4.4% of the saline control level, *p <* 0.05) (Figure 5A,B). In contrast, the hypoxia plus UFP-512 (1 mg/kg) hypoxia group showed a significantly increased CD11b expression after 3 and 10 days of hypoxic exposure (96.9% ± 3.3% and 94.3% ± 3.2% of the saline control level, *p <* 0.05) (Figure 5A,B) suggesting that UFP-512 attenuated the hypoxia-induced decrease in the expression of CD11b.

### 2.7. Effect of DOR Activation on Cortical pATF-1 and pCREB/CREB Expression

**Figure 6:**
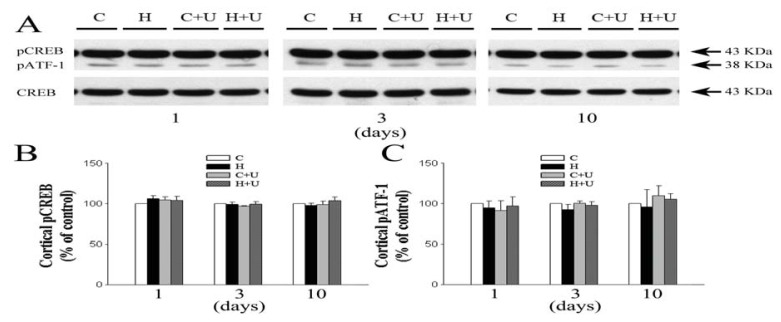
Effect of DOR activation on cortical pCREB and pATF-1 expression in hypoxia. (**A**) Representative blots of Western blot analysis of the cortical tissues; (**B**) Relative levels of pCREB. C, Relative levels of pATF-1; (**C**) Normoxic control. H, Hypoxia. C + U (UFP-512), DOR activation with UFP-512 treatment under normoxic conditions. H + U (UFP-512), DOR activation with UFP-512 treatment under hypoxic conditions. *N =* 4, 5 and 6 for 1, 3 and 10 days groups, respectively. Note that neither hypoxia nor DOR activation had any significant effect on the expression of pCREB and pATF-1 in the cortex.

Since DOR and BDNF mediated actions in the brain might involve CREB [21–23], we further explored the role of CREB as well as pATF-1 in the DOR-induced regulation of BDNF-TrkB signaling by determining their expression in the same brain tissues. Surprisingly, we found no significant alteration in pATF-1 or total/phosphorylated CREB in response to 1, 3 or 10 days hypoxia (pATF-1: 94.7% ± 8.4%, 92.5% ± 6.4% and 95.7% ± 21.6% of the control level respectively, *p >* 0.05; pCREB/CREB: 106.3% ± 3.7%, 99.1% ± 2.8% and 97.7% ± 2.8% of the control level respectively, *p >* 0.05). DOR activation also had no appreciable effect on the expression of these proteins (*p >* 0.05) (Figure 6).

## 3. Discussion

We report three major observations in the cortex exposed to hypoxia: (1) Hypoxia-induced damage is dependent on hypoxic duration, since prolonged (3- and 10-day) hypoxia caused a significant reduction in BDNF expression in the cortex when compared to short-term (1 day) hypoxia, and this reduction was partially reversed following DOR activation; (2) DOR activation partially reversed the hypoxia-induced reduction in functional TrkB (140-kDa) expression and attenuated the hypoxic increase in truncated TrkB (90-kDa) in the cortex exposed to 3- or 10-day hypoxia; (3) prolonged hypoxia (10 days) significantly upregulates the expression of TNF-α in the cortex but down-regulates the CD11b expression that was completely reversed following DOR activation. In addition, we observed that the levels of pCREB and pATF-1 were not significantly altered in the hypoxia-exposed cortex. These results support our hypothesis that the DOR-induced neuroprotection is mediated by an underlying BDNF-TrkB signaling pathway. Prolonged hypoxia causes a down-regulation of BDNF-TrkB signals leading to an increase in TNF-α in the cortex, while DOR activation upregulates BDNF-TrkB signals thereby decreasing the level of TNF-α in the hypoxic cortex.

This is the first study that demonstrates partial reversal of hypoxia-induced reduction of BDNF and functional TrkB expression as well as hypoxia-induced increase in truncated TrkB (90-kDa) and TNF-α expression in the cortex following DOR activation without any change in DOR density. DOR expression plays an important role in protection against cerebral hypoxia. Our previous studies have demonstrated that cortical DOR activation is protective against hypoxia and glutamate excitotoxicity in neurons [1,24]. DOR is an oxygen-sensitive protein and its expression depends on the duration and extent of hypoxia or ischemia. For example, DOR expression is upregulated by short-term or mild hypoxia, and downregulated after prolonged and severe hypoxia/ischemia [3] and other laboratories have confirmed the role of DOR in neuroprotection [25]. The findings of the present work further suggest that hypoxic stress affects the interaction between endogenous ligands and DOR (though there is no change in the expression of DOR), and disrupts the expression of BDNF and its functional receptor, TrkB, therefore leading to neuronal injury. The external addition of DOR agonists restores DOR signaling thereby reversing the effect of hypoxic insults on BDNF and its receptor.

BDNF is an important neuroprotective factor that has been proven to increase neuronal tolerance against hypoxia/ischemia in both *in vitro* and *in vivo* studies. A blockade of endogenous BDNF activity exacerbates the effects of cerebral ischemia [26]. Furthermore, an up-regulation of BDNF mRNA expression following DOR activation has been reported in animal models of depression [17]. BDNF binds specifically to TrkB whose density reaches adult levels at P30 [9]. TrkB has two major types of isoforms: a full-length TrkB receptor that possesses a tyrosine kinase domain, and a truncated isoform (90 kDa) that lacks a functional domain [9]. The proportion of truncated isoform usually increases following injury [11]. Our study suggests that TrkB is sensitive to hypoxic stress and the increase or decrease in its truncated isoform may play a crucial role in the mechanisms of neuronal injury and protection under hypoxic/ischemic condition. On the other hand, we demonstrated that DOR activation partly decreased the expression of 90 KDa TrkB after 3–10 days of hypoxia and upregulated the expression of 140 kDa TrkB. Therefore, we believe that DOR activation protects both ligand (BDNF) and receptor (140 kDa TrkB) for BDNF signaling in the brain. These observations led us to hypothesize that DOR-induced neuroprotection against hypoxia/ischemia is mediated by the BDNF-TrkB pathway, thus representing a novel signaling mechanism in DOR-mediated neuroprotection.

Brain injury is influenced by multiple factors, including stress duration and neuronal age [27,28]. The present work demonstrates that prolonged (3–10 days), but not short-term (1 day), hypoxia causes a progressive reduction in the cortical BDNF expression. On the other hand, in the hippocampus – a highly hypoxia-sensitive region of the brain, a single days’ exposure to hypoxia was enough to reduce BDNF expression. Interestingly, after 3- and 10-days of hypoxic exposure, BDNF expression did not decrease anymore. BDNF is widely distributed in the adult rodent brain with the highest levels in the cortex. It is mainly derived from the cerebral cortical neurons and projects to the hippocampus [29]. Indeed, evidence shows that BDNF produced in the entorhinal cortex is trafficked anterograde to the hippocampus [26]. Therefore, it is likely that 1-day hypoxia decreases BDNF levels in the hippocampus but not in the cortex, which is a relatively hypoxia-tolerant region. With an increase in the duration of hypoxia, the cortical BDNF is released and partially transported to the hippocampus as a compensatory mechanism to rescue it from the hypoxic insult. Therefore, cortical BDNF levels decrease while hippocampal BDNF levels are less affected after 3–10 days of hypoxic exposure as illustrated in the present work. Since the cortex has a rich distribution of DOR, the hypoxia-induced reduction of BDNF in this region can be reversed/attenuated by increasing DOR activity through external administration of DOR agonists.

In a sharp contrast, the subcortical regions and cerebellum did not show any significant alteration in BDNF expression under hypoxic exposure for various periods (1, 3 and 10 days). This is partially because the subcortical and cerebellar regions may be relatively less sensitive to hypoxia and their BDNF can be compensated by transportation along cortical, nigral and brainstem neurons during hypoxia [30–32]. In fact, the anterograde transport of BDNF via the cortical and nigral neurons accounts for most of the BDNF in the adult rat subcortex [31,32]. Similarly, the cerebellar neurons partly contribute to the total cerebellar BDNF syntheses, as the remaining BDNF is obtained from other sources such as the inferior olivary nucleus in the brainstem [30]. With these supplemental mechanisms, the subcortical and cerebellar regions can sustain hypoxic stress in terms of BDNF expression.

Although neurons are the chief source of BDNF, it may also come from the microglia. Microglia are immune cells present in the central nervous system and activated in response to injury and diseases and are known to produce neurotrophic factors such as BDNF, glial cell line-derived neurotrophic factor (GDNF), basic fibroblast growth factor (bFGF) and vascular endothelial growth factor (VEGF), which provide trophic support to neurons during injury [33]. Pioneering antibody research identified the integrin beta 2 protein (also called ITGB2) as one of the key proteins in the microglial recruitment [34]. The ITGB2 subunit A, commonly referred to as CD11b or OX42, and the antibodies against this subunit are widely used as microglial markers. It recognizes the microglia in both the resting and the activated states [35]. Microglial activation labeled by OX42 was highly expressed in white matter regions such as the corpus callosum, external capsule, and internal capsule compared with sham-operated control rats, other than in the gray matter of rat brains [36]. We found that DOR activation partially reversed the hypoxia-induced decrease in cortical CD11b protein levels after 3–10 days of hypoxia. This phenomenon is consistent with the change in BDNF under hypoxia, suggesting that a prolonged (3–10 days) hypoxia may injure microglia, thus partially contributing to the reduction of BDNF in the cortex. DOR partially reverses such injury. Since BDNF inhibits microglia apoptosis under inflammatory conditions [37], the DOR-induced BDNF expression may generate a protective signal for the microglia, thereby preventing microglia injury and maintaining the level of BDNF.

However, activated microglia exerts both beneficial and deleterious effects on neurons. For example, activated microglia following cerebral ischemia express a variety of proinflammatory cytokines including interleukin-1β (IL-1β), interleukin-6 (IL-6) and tumor necrosis factor-α (TNF-α), which induce neuroinflammation and neurotoxicity [33]. The available evidence indicates that microglia activation results in TNF-α expression in hypoxic/ischemic brain depending on the duration of hypoxia or ischemia [38,39]. The overexpression of TNF-α is toxic to the neurons [40] and may account for the pathophysiology of some neurodegenerative disorders. For example, in a model of multiple sclerosis, it is demonstrated that BDNF decreases TNF-α expression and upregulates IL-10 expression, an anti-inflammatory cytokine [19]. In an experimental stroke model, intranasal BDNF was shown to modulate local inflammation and protect against cerebral ischemia by upregulating anti-inflammatory cytokine IL-10, down-regulating the pro-inflammatory cytokine TNF-α, and in turn increasing the DNA-binding activity of NFKB [41]. In the present study, we observed that TNF-α protein expression tended to increase after 3 days of hypoxic exposure, and a significant increase was detected after 10 days of hypoxic exposure. The loss of BDNF during this hypoxic period could also be partially attributed to the deleterious microglial environment owing to a marked increase in the TNF-α expression.

The BDNF gene is one of the targets of CREB pathway [42]. The DOR-mediated changes in BDNF can be linked to CREB phosphorylation. A previous study demonstrated that DOR activation induces a dose-dependent increase in CREB phosphorylation and that this effect was reversed by Naltrindole [23]. Indeed, our recent studies in 293 cells show that DOR activation increases CREB phosphorylation (data unpublished). We therefore speculated that the DOR-mediated upregulation of BDNF-TrkB in the hypoxic brain utilizes a CREB pathway. However, we did not observe any appreciable alteration in the levels of pCREB and pATF-1 in the cortex, either in normoxia, hypoxia and/or with DOR activation. We used the well-documented CREB specific antibodies (Millpore, 06-519) [43] that recognize both p43-phosphorylated-CREB and phosphorylated-ATF-1, another CRE-binding protein structurally similar to CREB with an identical amino-acid sequence especially in the phosphorylation domain. Since we used the same tissues, even the same protein samples for different assays, these results further demonstrate that the above changes in BDNF, TrkB, TNF-α and CD11b are specific. However, it needs more investigations to clarify if CREB and ATF-1 are differentially involved in DOR signaling under different conditions and in various tissues/cells.

**Figure 7:**
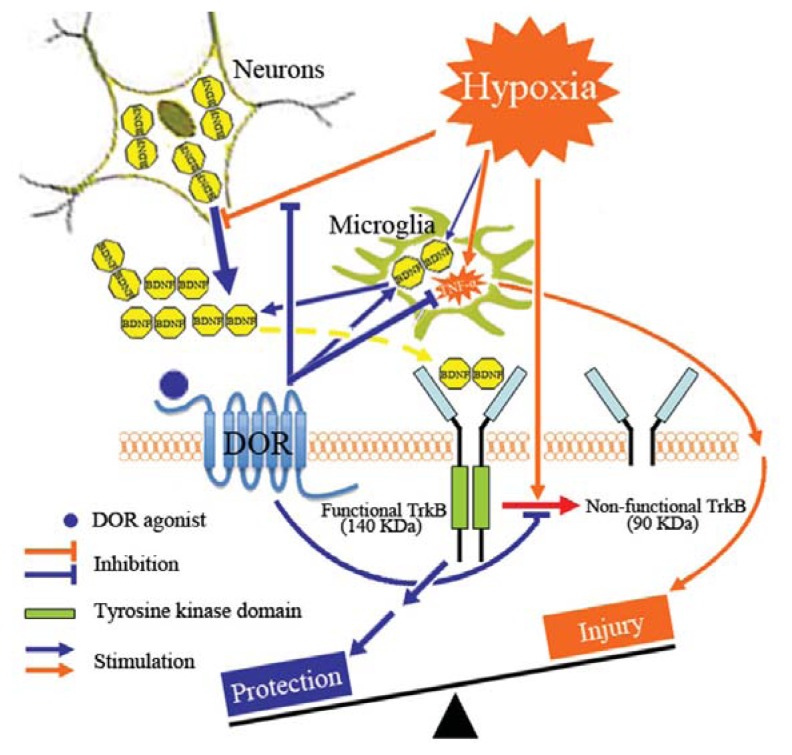
Schematic demonstration of the DOR-BDNF-TrkB pathway in brain protection against hypoxic injury. Prolonged hypoxia causes a down-regulation of BDNF-TrkB signals resulting in an increased cortical TNF-α, while DOR activation up-regulates BDNF-TrkB signals thereby decreasing the level of TNF-α in the hypoxic cortex.

To summarize, we propose a potential neuroprotective mechnism involving interactions among DOR, BDNF-TrkB and TNF-α that is schematically illustrated in Figure 7. Briefly, prolonged hypoxia causes a down-regulation of BDNF-TrkB signals in the cortical region resulting in an increased cortical TNF-α expression, while DOR activation upregulates BDNF-TrkB signals thereby decreasing the level of TNF-α and protecting the cortex against hypoxic injury. However, further investigations are need to verify this hypothesis, especially for delineating the precise role of DOR in this meachanistic pathway.

## 4. Experimental Section

### 4.1. Animals and Hypoxic Exposure

Male C57BL/6 mice (Charles River Laboratories, Wilmington, DE, USA) at the age of 30-day old (P30) were maintained under a 12 h light/12 h dark cycle and 22 °C with standard rodent chow *ad libitum*. All animal procedures were approved by the University of Texas medical school at Houston Animal Care and Use Committee (Animal Welfare Assurance Number: HSC-AWC-10-144) and conformed to the National Institutes of Health Guide for the Care and Use of Animals in Research.

In total, 60 mice were randomly divided into 4 groups: (1) Normoxia control with saline (*n =* 15); (2) Hypoxia with saline (*n =* 15); (3) DOR activation with UFP-512 treatment under normoxic conditions (*n =* 15); and (4) DOR activation with UFP-512 treatment under hypoxic conditions (*n =* 15). Saline or UFP-512 (1 mg/kg) was daily administrated by intraperitoneal injection. In the hypoxic mice, the first injection was administered at 30-min prior to hypoxia induction. The numbers of the animals used for 3 time points (1-, 3- and 10-day) were 4, 5, and 6 respectively in each group under 4 experimental conditions (*i.e.*, normoxia with saline, hypoxia with saline, normoxia with UFP-512, hypoxia with UFP-512).

To expose mice to hypoxic stress, 2 cages of mice (2 or 3/cage) were placed in a plexiglass chamber (30″W x 20″D x 20″H) (Biospherix, Redfield, NY, USA). The chamber was connected with the outside environment via holes in the wall of the chamber; therefore, CO_2_ levels and humidity in the chamber were kept constant as the ambient levels. O_2_ levels in the chamber were strictly kept at (10 ± 0.5)% by constantly flushing with nitrogen that was automatically controlled by a ProOx P110 Oxygen Controller with E702 Oxygen Sensor (Biospherix, Redfield, NY, USA). The ProOx O_2_ controller has an extremely wide range from 0.1% O_2_ all the way up to 99.9% O_2_ with the variation of 0.1%, which provides a very important tool for our research in terms of accurate control of O_2_ level. The mice were exposed to hypoxia for 1, 3 or 10 days. For both the saline control and UFP-512 control groups, mice were treated in the similar fashion as the hypoxic exposure mice, but under normoxia (21% of O_2_ level) instead of hypoxia.

The chamber used was custom designed, based on the experimental demands and was fitted with two eight-inch iris ports to access the animals during a prolonged hypoxic exposure in order to keep exposure changes to a minimum. Our daily saline or UFP-512 administration was carried out in the hypoxic chamber by accessing the animals with hands stretching through the iris ports in the front wall of the chamber. Therefore, all the procedures were uniform and strictly controlled for hypoxic exposure.

### 4.2. Chemicals and Reagents

UFP-512 (H-Dmt-Tic-NH-CH(CH2–COOH)-Bid), a specific and potent DOR agonist [44–46], was synthesized by our research team. UFP-512 was dissolved in water with a stock concentration of 10 mM, and stored at −80 °C. Immediately before use, the stock solution was diluted in saline to the expected concentrations. The doses of UFP-512 was 1 mg/kg based on the previous works [47,48].

Rabbit polyclonal anti-DOR and anti-phospho-CREB (Ser^133^) were obtained from Millpore (Cat: AB1560, 06-519). Rabbit monoclonal anti-CREB and TrkB was obtained from Cell Signaling Technology (Cat: 9197S, 4603S). Mouse monoclonal anti-TNF alpha and rabbit polyclonal anti-CD11b was obtained from Abcam Inc (Cat: ab1793, ab75476). Rabbit polyclonal anti-BDNF was purchased from Alomone Labs Ltd (Cat: ANT-010). Mouse monoclonal anti-β-actin and RIPA buffer were obtained from Sigma-Aldrich (Cat: A5441, R0278). Horseradish peroxidase conjugated goat anti-rabbit/mouse IgG (H + L) were purchased from Invitrogen (Cat: G-21234, G-21040). Laemmli sample buffer, Precision Plus Protein™ Dual Color Standards and mini-protean precast gels were obtained from Bio-rad (Cat: 161-0737, 161-0374, 456-1083).

### 4.3. Western Blot Analysis

Mice were sacrificed at day 1, 3 or 10, and the brain was removed quickly. The cortical, subcortical (mainly striatum and thalamus), cerebellar and hippocampal tissues were dissected on ice, frozen immediately on dry ice, and then stored at −80 °C until use. The tissues were ultrasonically homogenized in a RIPA buffer and protease inhibitor cocktail, and the homogenates were centrifuged at 12,000× *g* for 10 min at 4 °C. Protein concentrations were determined with a protein assay kit (Bio-Rad, Hercules, CA, USA). The supernatants of tissue homogenates (40 μg protein equivalent each) of the cortex, subcortex, cerebellum and hippocampus from each mouse were boiled at 100 °C in laemmli sample buffer (Bio-Rad) for 5 min. After, the samples were electrophoresed on 4%–15% mini-protean precast gels (Bio-Rad), and transferred to 0.20 μm nitrocellulose membrane (Whatman). Membranes were blocked with 5% (*m*/*v*) nonfat dry milk in 0.1% Tween 20 (TBS-T; 2 mmol/L Tris-HCl, 50 mmol/L NaCl, pH 7.4) for 1 h at room temperature and subsequently incubated overnight at 4 °C in the blocked buffer with the p-CREB (1:1000), CREB(1:2000), CD11b(1:800), TrkB (1:1000), TNF alpha (1:1000) and BDNF (1:200) antibodies, respectively. The membranes were washed with 0.1% Tween 20, and then treated with horseradish peroxidase-conjugated anti-rabbit and anti-mouse IgG (1:5000; 1:10,000) for 1 h at room temperature. Peroxidase activity was visualized with a super signal West Pico Chemiluminescent Substrate (Thermo Scientific, Cat: 34079). Stripping filters and reprobing for β-actin were carried out for normalization. Controls for nonspecific binding were determined by omission of the primary antibody. The proteins were quantified by measuring optical densities of immunostained bands using an image analysis system (Image J; NIH, Bethesda, MD, USA).

### 4.4. Statistical Analysis

Two independent and blinded investigators examined the end point assessments. Data was expressed as mean ± SEM. One-way ANOVA with the post hoc test of Least Significant Difference (LSD) was used to analyze the difference between various groups. Statistical significance was determined based on *p* values < 0.05.

## 5. Conclusions

Our data suggest that the cortical neurons and microglia lose their ability to maintain normal levels of BDNF and functional TrkB after a prolonged exposure to hypoxia. Therefore, both BDNF and functional TrkB protein levels decrease, along with a significant increase in the level of TNF-α expression following a severe hypoxic injury. DOR activation can partially reverse the hypoxia-induced decrease in the BDNF and functional TrkB levels and protect the brain from hypoxic/ishcmic injuury. The upregulation of BDNF along with its TrkB receptor and CD11b *versus* the downregulation of the pro-inflammatory cytokine, TNF-α, suggests their role in the mechanism of DOR-mediated neuroprotection against hypoxia. Targeting the DOR-BDNF-TrkB pathway provides a potential therapy for neuroprotection against hypoxic/ischemia encephalonpathy. However, further studies are needed to ascertain the mechanisms of DOR-mediated regulation of BDNF-TrkB and TNF-α under hypoxic conditions.

## Abbreviations

DOR: delta-opioid receptor
BDNF: brain-derived neurotrophic factor
TrkB: tyrosine kinase B
TNF-α: tumor necrosis factor α
CREB: cyclic AMP response element binding protein
p-CREB: CREB phosphorylation
